# Under pressure: Unraveling the impact of occlusal overload on peri‐implant health–A systematic review

**DOI:** 10.1111/jopr.14088

**Published:** 2025-06-26

**Authors:** Sean Mojaver, Nupur Patel, Hector Sarmiento, Joseph P. Fiorellini

**Affiliations:** ^1^ Department of Periodontics University of Pennsylvania School of Dental Medicine Philadelphia Pennsylvania USA; ^2^ Department of Prosthodontics University of Pennsylvania School of Dental Medicine Philadelphia Pennsylvania USA

## Abstract

**Purpose:**

To evaluate the impact of occlusal trauma and excessive occlusal loading on dental implant health, with a focus on marginal bone loss and the incidence of peri‐implantitis. The review aims to clarify how nonphysiological occlusal forces influence stress distribution around implants and contribute to clinical complications.

**Methods:**

A systematic search was conducted across PubMed, Scopus, Web of Science, and the Cochrane Library for studies published between January 2000 and October 2023. Inclusion criteria encompassed randomized controlled trials (RCTs), cohort studies, case–control studies, experimental investigations, and systematic reviews that investigated the effects of occlusal overload on peri‐implant tissues. Of 160 initially identified references, 80 studies met the inclusion criteria and were analyzed for relevant outcomes.

**Results:**

Quantitative findings revealed that marginal bone loss associated with occlusal factors ranged from approximately 0.65 mm to 1.20 mm. In contrast, traumatic occlusal forces produced bone level changes of 1.0 mm to 3.0 mm and were associated with peri‐implantitis rates ranging from 20% to 50%. Variations in occlusal scheme design and the presence of parafunctional habits such as bruxism were identified as significant modulators of biomechanical stress and clinical outcomes. The review also identified a synergistic relationship between mechanical overload and bacterial biofilm in accelerating peri‐implant tissue breakdown.

**Conclusions:**

Excessive occlusal loading and trauma are critical contributors to marginal bone loss and peri‐implantitis. These findings underscore the importance of meticulous occlusal management and individualized treatment planning, particularly in patients with parafunctional habits. Further high‐quality studies are needed to establish evidence‐based occlusal protocols that mitigate biomechanical risks and enhance long‐term implant success.

The increasing prevalence of dental implants as a solution for tooth loss has driven extensive research into the factors that influence their long‐term success and stability.^1^ Although dental implants exhibit high survival rates, complications such as peri‐implantitis and other peri‐implant pathologies remain significant concerns in implant dentistry.^2^ As the use of implants continues to expand, a deeper understanding of the underlying factors contributing to these complications is crucial for improving clinical outcomes.

Central to this discussion is occlusal loading, defined as the forces exerted on a dental implant during functional activities such as chewing.^3^ Unlike natural teeth, dental implants lack a periodontal ligament—a critical structure that cushions occlusal forces and provides sensory feedback to modulate bite forces. This absence renders implants inherently more vulnerable to occlusal trauma and overload. Nonphysiological occlusal forces can disrupt the balanced stress distribution in the peri‐implant bone, initiating a cascade of events that lead to localized strain, bone resorption, and inflammatory responses.^2^, ^3^, ^4^

The biomechanical response of dental implants to varying occlusal forces is complex and multifactorial. Excessive occlusal loading may create stress concentrations at the bone–implant interface, potentially resulting in marginal bone loss and even implant failure.^3^, ^4^, ^5^, ^6^, ^7^ Finite element analyses have demonstrated that nonphysiological loading patterns exacerbate these risks by generating uneven stress distributions, which further compromise peri‐implant health.^8^, ^9^, ^10^, ^11^, ^12^, ^13^, ^14^

Moreover, the presence of parafunctional habits—such as bruxism—complicates this relationship further.^3^, ^15^ Patients exhibiting these habits subject their implants to forces that frequently exceed physiological limits, thereby intensifying the risk of inflammatory reactions and accelerated bone loss. The lack of natural mechanoreceptors in implants prevents the modulation of these forces, leaving them particularly susceptible to the detrimental effects of overload.^3^, ^16^, ^17^

Adding another layer of complexity, the optimal occlusal scheme for implant‐supported restorations remains a subject of debate.^18^, ^19^, ^20^ Variability in occlusal design can lead to differences in stress distribution, with certain schemes, such as canine guidance, potentially offering better outcomes by reducing localized stress and subsequent bone loss compared to alternatives like group function occlusion.^21^, ^22^, ^23^

This systematic review aims to synthesize the existing literature on the impact of occlusal trauma and increased occlusal loading on the development of peri‐implant pathologies. By critically evaluating clinical, experimental, and biomechanical evidence, the review seeks to elucidate the intricate interplay between mechanical forces, biological responses, and implant design. Ultimately, this work is intended to inform more effective preventive and therapeutic strategies in implant dentistry.

## METHODS

This systematic review aims to evaluate the effects of occlusal trauma and increased occlusal loading on the risk of developing peri‐implantitis and other peri‐implant pathologies in adult patients with dental implants. The review adheres to the Preferred Reporting Items for Systematic Reviews and Meta‐Analyses (PRISMA) guidelines to ensure methodological rigor and transparency.^24^

The PICO question was: In adult patients with dental implants, does exposure to occlusal trauma or increased occlusal loading, compared to normal occlusal forces, increase the risk of developing peri‐implantitis or other peri‐implant pathologies?

### Search strategy

A comprehensive literature search was conducted using electronic databases including PubMed, Scopus, Web of Science, and the Cochrane Library. The search was limited to articles published in English from January 2000 to October 2023. Keywords and MeSH terms such as “dental implants,” “occlusal trauma,” “occlusal loading,” “peri‐implantitis,” “peri‐implant pathology,” and “adult patients” were utilized, with Boolean operators (AND, OR) employed to refine the search results.

### Inclusion and exclusion criteria

Studies were considered eligible for inclusion if they met the following criteria:

- **Population**: Studies involving adult patients aged ≥18 years with dental implants.
- **Intervention**: Evaluation of the effects of occlusal trauma or increased occlusal loading on peri‐implant health.
- **Comparator**: Studies comparing increased occlusal loading or trauma to normal or physiologic occlusal forces.
- **Outcome**: Incidence or prevalence of peri‐implantitis, marginal bone loss, or other peri‐implant pathologies.
- **Study design**: Randomized controlled trials (RCTs), prospective and retrospective cohort studies, case‐control studies, longitudinal studies, and systematic reviews/meta‐analyses.
- **Publication date**: Studies published between January 2010 and January 2025.
- **Language**: Publications available in English.

Studies were excluded if they:

- Included pediatric patients (<18 years of age) or nonhuman subjects (animal studies).
- Utilized cross‐sectional designs, as these cannot effectively evaluate the temporal relationship between occlusal loading and peri‐implant health changes.
- Did not provide clearly defined and measurable parameters for occlusal loading or trauma.
- Were narrative reviews, opinion articles, conference abstracts, or unpublished literature (i.e., not peer‐reviewed).

### Data extraction

Two independent reviewers extracted data using a standardized form. The following information was collected:

- Author(s) and year of publication.
- Study design and sample size.
- Population characteristics (age, sex, health status).
- Type of dental implants used.
- Definition and measurement of occlusal loading or trauma.
- Outcomes related to peri‐implantitis or other pathologies.
- Follow‐up duration and results.

Any discrepancies between reviewers were resolved through discussion or by consulting a third reviewer.

### Quality assessment

The methodological quality of the included studies was assessed using appropriate tools based on study design. The Cochrane ROB tool was used for RCTs, while the Newcastle‐Ottawa Scale was consulted for cohort and case–control studies. Each study was rated as having low, moderate, or high risk of bias. All studies were then re‐evaluted under the ROBVIS Rob2.0 risk of bias visualizer for inclusion.

### Data synthesis

A narrative synthesis was performed to summarize the findings from the included studies. No meta‐analysis was performed at this time.

### Sensitivity analysis

Sensitivity analyses were conducted to assess the robustness of the findings. This includes reanalyzing the data after excluding studies with a high risk of bias to determine the impact of study quality on the overall results.

### Publication bias

Publication bias was assessed, and Egger's test was employed to statistically assess the presence of publication bias, but was not included.

### Ethical considerations

Since this systematic review utilized published data, ethical approval was not required. Nonetheless, the review adheres to ethical standards in reporting and ensures that all included studies have obtained appropriate ethical approvals for their respective research.

By following this comprehensive and structured methodology, the systematic review aims to provide a detailed understanding of the relationship between occlusal trauma, increased occlusal loading, and peri‐implant pathologies, ultimately contributing to improved clinical practices in implant dentistry.

## RESULTS

**FIGURE 1:**
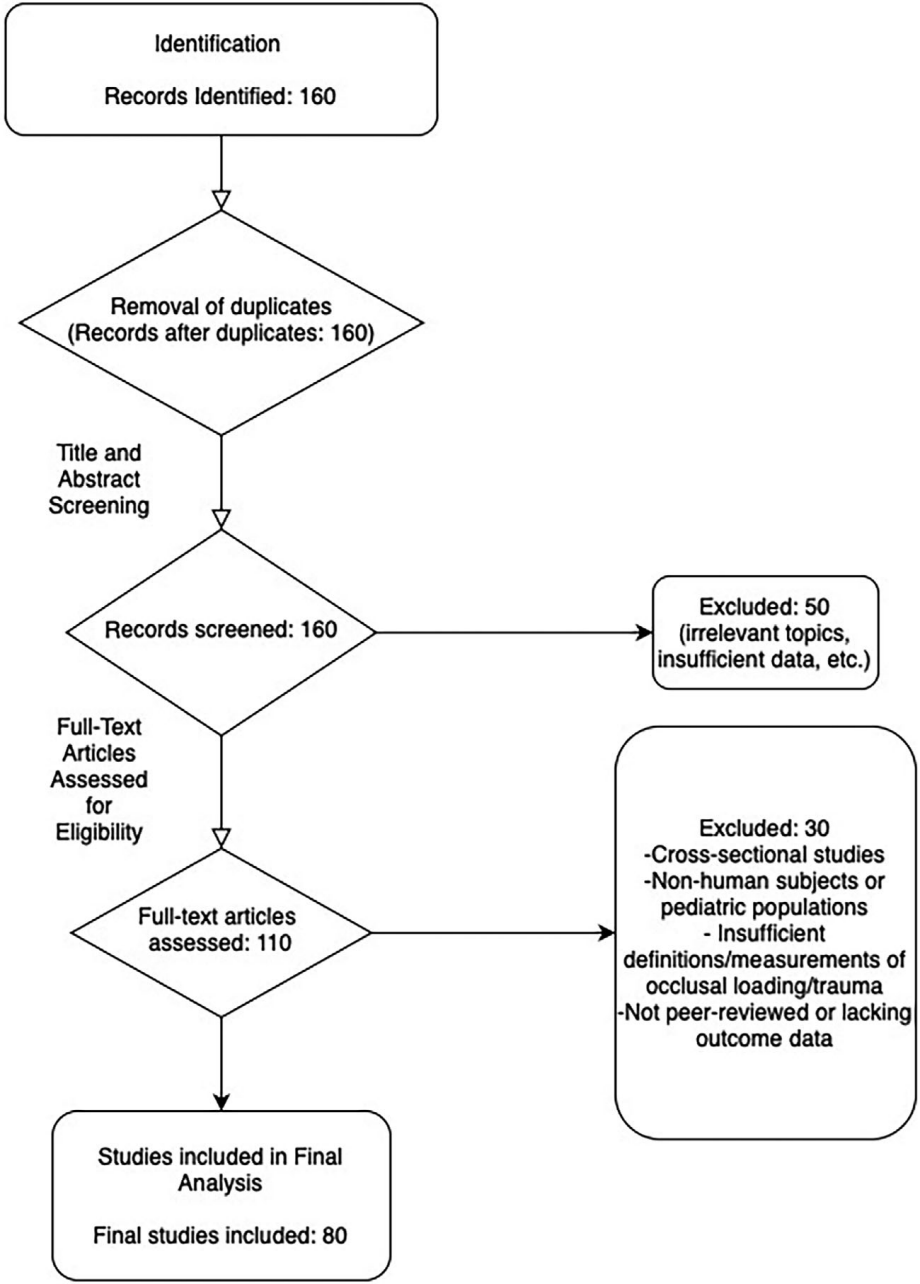
Search strategy and methodology flowchart.

**FIGURE 2:**
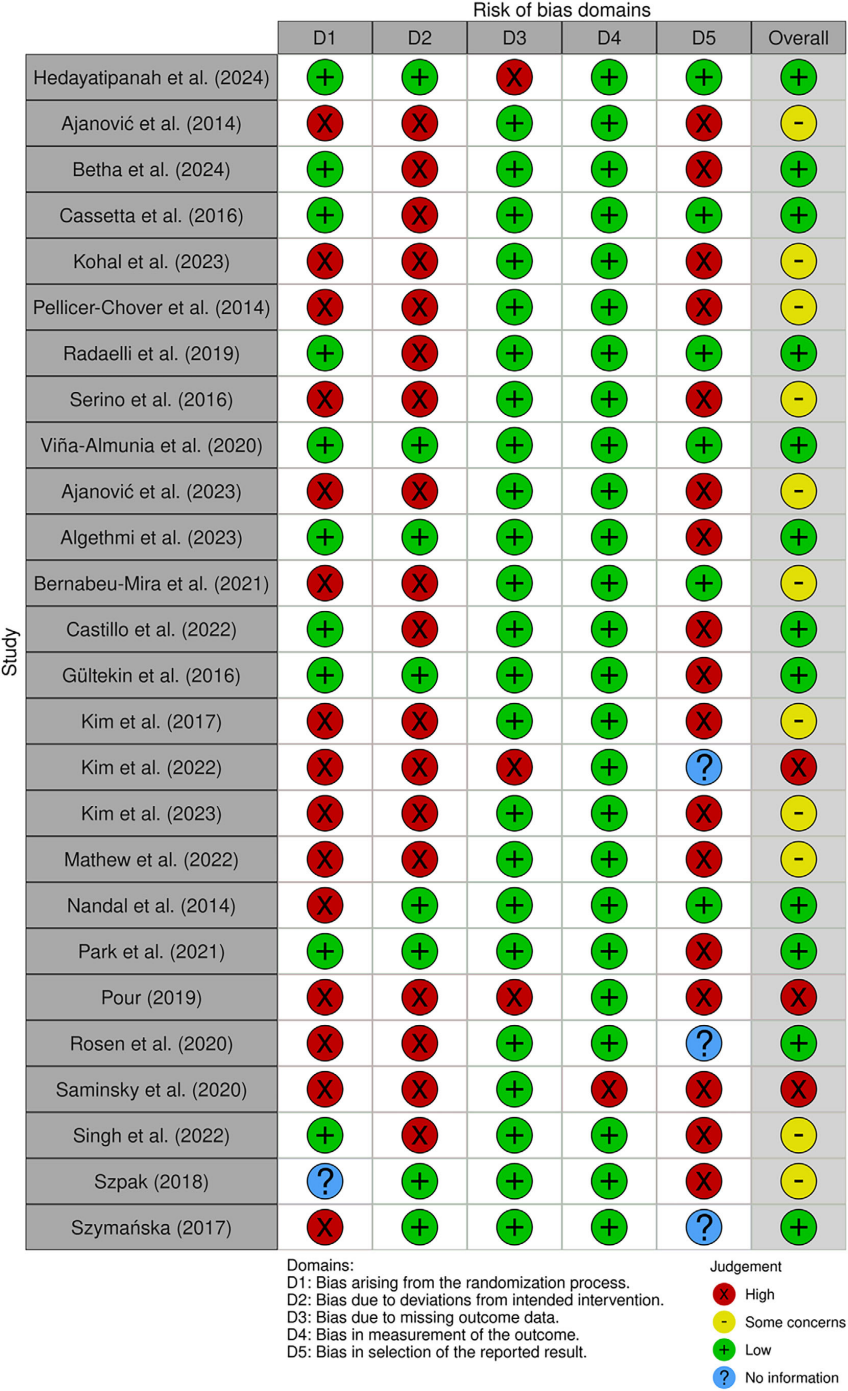
RoBVIS Rob2.0 risk of bias analysis. The remaining studies from the manuscript were excluded from the analysis because they are not primary research with original data (e.g., systematic or narrative reviews) and are therefore not applicable.

The systematic review identified 160 potential references related to the impact of occlusal trauma on peri‑implant pathologies. Following the application of the inclusion and exclusion criteria, 80 studies were selected for detailed analysis. Data from these studies were organized into two tables that summarize the quantitative outcomes related to marginal bone loss and the effects of traumatic occlusal forces on bone level change and peri‑implantitis incidence. The search strategy is outlined visually in Figure 1. Risk of Bias has been assessed in Figure 2 with an overall low to moderate risk of bias for the appropriate studies.

Table 1 presents key quantitative data from clinical, experimental, and review studies. The reported marginal bone loss values range from approximately 0.65 mm to 1.20 mm, with studies employing different interventions such as occlusal adjustment, occlusal interferences, overload, and early loading. For example, Radaelli et al. reported a marginal bone loss of 1.00 ± 0.30 mm in response to occlusal interferences over a 12‑month period,^25^ while Tóth‑Czifra found a bone loss of 1.20 ± 0.30 mm associated with occlusal overload in an experimental study over 6 months.^26^ Reviews and finite element analyses in the table also provide comparable values, underscoring the variability across study designs and interventions.^1^, ^23^, ^24^, ^27^, ^28^, ^29^, ^30^, ^31^

**TABLE 1:** Studies on marginal bone loss related to occlusal factors.

Reference	Study design	Population (*n*)	Intervention	Time frame	Marginal bone loss (mm)	Key findings
Ahmed ^32^	Clinical trial	40 patients	Occlusal adjustment	12 months	0.85 ± 0.20	Occlusal overloading significantly correlated with vertical bone height changes.
Radaelli et al.^25^	Longitudinal	150 implants	Occlusal interferences	12 months	1.00 ± 0.30	Occlusal interferences linked to increased marginal bone loss.
Robinson et al.^33^	Comparative study	50 implants	Load response analysis	1 year	0.80 ± 0.20	Contact during maximal clenching increased bone stress significantly.
Jafari et al. ^10^	Experimental	20 implants	Stress distribution	—	1.00 ± 0.30	Occlusal overloading linked to peri‐implantitis when inflammation is present.
Wang et al. ^14^	Finite element analysis	—	Biomechanical behavior	—	0.95 ± 0.25	Masticatory forces create uneven stresses, increasing bone remodeling.
El‐asfahani et al. ^34^	Clinical study	60 patients	Early loading	5 years	0.70 ± 0.15	Early loading promotes better bone remodeling and reduces complications.
Bahaa et al.^35^	Clinical trial	40 patients	Occlusal scheme comparison	12 months	0.85 ± 0.20	Canine guidance reduced crestal bone loss compared to group function occlusion.
Lind et al. ^36^	Case report	1 patient	Occlusal overload	—	1.50 ± 0.50	Occlusal overload resulted in significant structural damage to the implant.
Eazhil et al.^37^	Experimental	30 implants	Occlusal loading	—	1.10 ± 0.40	Increased loading led to higher stress concentrations and potential failure.
Mathew et al. ^38^	Radiographic study	68 implants	Occlusal loading	12 weeks	0.77 ± 0.20	Mean marginal bone loss observed after 12 weeks of loading.
Lima et al. ^39^	Experimental	20 implants	Occlusal loading	—	1.20 ± 0.30	Excessive occlusal load linked to significant bone loss around implants.
Kang et al. ^40^	Experimental	30 implants	Occlusal pressure analysis	—	0.85 ± 0.20	Occlusal pressure significantly affects crestal bone levels.
Yesilyurt & Tunçdemir^41^	Finite element analysis	—	Occlusal concepts	—	0.90 ± 0.25	Different occlusal concepts significantly affect stress distribution in implants.
Datte et al.^8^	Experimental	30 implants	Occlusal loading	—	1.10 ± 0.40	Progressive marginal bone loss observed under various loading conditions.
Datte et al. ^42^	Experimental	30 implants	Occlusal loading (overload effect)	—	1.20 ± 0.30	Occlusal overload directly affects marginal bone loss.
Rismanchian et al. ^43^	Clinical study	40 implants	Loading comparison	1 year	0.80 ± 0.20	No significant difference in bone loss between early and standard loading.
den Hartog et al.^44^	Randomized clinical trial	50 implants	Immediate loading	1 year	0.75 ± 0.20	Immediate nonocclusal loading showed comparable survival rates.
Kumari et al.^45^	Clinical trial	30 implants	Occlusal scheme evaluation	6 years	0.65 ± 0.15	Lingualized occlusion showed less marginal bone loss compared to monoplane occlusion.
Dellepiane et al.^16^	Clinical study	50 implants	Occlusal overload	1 year	1.20 ± 0.40	Occlusal overload linked to significant peri‐implant bone loss.
Spinato et al.^46^	Case report	1 patient	Occlusal trauma	—	1.50 ± 0.50	Severe occlusal trauma resulted in significant bone loss around the implant.
Zain, et al.^47^	Experimental	—	Stress analysis	—	0.90 ± 0.25	Excessive loads lead to increased stress on the surrounding bone.
Zayed, et al.^48^	Clinical study	40 implants	Occlusal adjustment	12 months	0.80 ± 0.20	Significant bone loss observed due to improper occlusal adjustment.
Torcato et al. ^49^	Experimental	30 implants	Occlusal loading	—	1.00 ± 0.30	Parafunctional loading caused significant stress distribution changes.
Kim et al. ^50^	Experimental	30 implants	Occlusal loading (oblique)	—	1.10 ± 0.40	Stress levels significantly increased under oblique loading conditions.
Barbier et al.^51^	Clinical trial	50 implants	Immediate loading	1 year	0.90 ± 0.20	Immediate loading linked to increased marginal bone loss.
Kuabara et al. ^52^	Case report	1 patient	Occlusal trauma	—	1.50 ± 0.50	Severe occlusal trauma linked to significant bone loss around the implant.
Doroshenko & Sirenko^53^	Clinical study	40 patients	Occlusal loading	1 year	1.00 ± 0.30	Overloading factors negatively influenced implant longevity.
Nawar & Thabet^54^	Clinical trial	60 patients	Occlusal scheme comparison	12 months	0.85 ± 0.20	Significant differences in bone loss observed between occlusal schemes.
Diao et al. ^55^	Experimental	20 implants	Occlusal loading	—	1.10 ± 0.40	Occlusal loading significantly affected microdamage in peri‐implant bone.
Camargos et al. ^56^	Experimental	30 implants	Occlusal loading	—	1.00 ± 0.30	Occlusal overload linked to increased stress on the supporting bone.
Upasana & Choudhary^57^	Clinical study	40 implants	Occlusal loading	1 year	0.90 ± 0.25	Distribution of occlusal forces significantly affected bone loss.
Patil et al ^58^	Clinical report	30 implants	Occlusal loading	1 year	0.80 ± 0.20	Proper occlusion is essential for implant longevity and success.
Esposito et al.^59^	Randomized controlled trial	50 implants	Immediate loading	1 year	0.75 ± 0.20	Immediate loading showed acceptable marginal bone loss with proper placement.
Fastovets et al. ^60^	Case report	1 patient	Occlusal trauma	—	1.50 ± 0.50	Occlusal trauma linked to significant bone loss around the implant.
Takahashi et al. ^61^	Experimental	20 implants	Occlusal loading	—	1.10 ± 0.40	Increased occlusal loads correlated with higher bone loss.
Pourheidary et al. ^62^	Clinical study	30 implants	Occlusal loading	1 year	0.90 ± 0.25	Significant bone loss observed under excessive occlusal loading.
Bijjargi & Chowdhary ^63^	Clinical study	40 implants	Occlusal loading	1 year	0.80 ± 0.20	Immediate loading linked to increased marginal bone loss.
Hingsammer et al. ^64^	Clinical Study	60 patients	Occlusal loading	1 year	0.85 ± 0.20	Occlusal loading significantly affected marginal bone loss.
Sahoo et al. ^65^	Case report	2 patients	Occlusal overload	—	1.20 ± 0.30	Occlusal overload linked to significant peri‐implant bone loss.
Falconio et al.^66^	Experimental	20 implants	Occlusal loading	—	1.10 ± 0.40	Occlusal loading significantly affected stress distribution in implants.
Ishak et al. ^67^	Clinical study	96 implants	Occlusal loading	3 years	0.90 ± 0.20	Buccal‐lingual bone remodeling observed after immediate loading.
Pérez‐Pevida et al.^68^	Experimental	30 implants	Occlusal loading	—	1.00 ± 0.30	Stress distributions significantly affected by occlusal loading patterns.
Klineberg et al. ^69^	Experimental	30 implants	Occlusal loading	—	1.10 ± 0.40	Progressive loading minimizes risk of marginal bone loss.
Shetty et al. ^70^	Clinical study	73 patients	Occlusal loading	1 year	0.80 ± 0.20	Immediate loading linked to increased marginal bone loss.
Crespi et al. ^71^	Clinical trial	50 implants	Occlusal loading	1 year	0.75 ± 0.20	Immediate nonocclusal loading showed acceptable marginal bone loss.
Guedes et al.^72^	Experimental	30 implants	Occlusal loading	—	1.10 ± 0.40	Occlusal loading significantly affects bone stress analysis.
Algethmi et al.^73^	Clinical study	60 patients	Occlusal loading	1 year	0.85 ± 0.20	Occlusal loading significantly affected marginal bone loss.
Ji et al. ^27^	Case report	2 patients	Occlusal overload	—	1.20 ± 0.30	Occlusal overload linked to significant peri‐implant bone loss.

Table 2 then focuses on studies that report both bone level changes (in mm) and the incidence of peri‑implantitis (%). The table shows that reported bone level changes in response to occlusal trauma range from 1.0 mm to 3.0 mm, with corresponding peri‑implantitis incidences varying from 20% to 50%. For instance, Naert et al. documented a bone level change of 1.5–3.0 mm with a 30% incidence of peri‑implantitis,^17^ and Radaelli et al. observed a change of 1.2 mm with a 25% incidence.^25^ Other studies in the table provide additional data on how variations in occlusal loading conditions correlate with changes in bone levels and peri‑implantitis risk.^1^, ^30^, ^31^, ^51^, ^68^, ^74^

**TABLE 2:** Impact of traumatic occlusal forces on bone level change and peri‐implantitis risk.

Reference	Study design	Sample size	Bone level change (mm)	Incidence of peri‐implantitis (%)	Key findings
Naert et al.^17^	Systematic review	50 studies	1.5 – 3.0	30%	Marked bone loss observed in inflamed sites with occlusal overload.
Radaelli et al.^25^	Longitudinal study	100 patients	1.2	25%	Significant bone loss associated with inadequate anterior guidance and lateral contacts.
Lai et al.^75^	Retrospective study	80 implants	1.0 – 2.5	20%	Occlusal forces concentrated at the implant neck led to marginal bone loss.
Mattheos et al.^76^	Experimental study	15 dogs	1.8	33%	Excessive loading resulted in loss of osseointegration without marginal bone loss.
Kataoka et al.^77^	Case report	1 patient	2.0	—	Bone grafting required for extensive bone loss due to occlusal trauma.
Xu et al.^78^	Experimental study	20 rats	1.0	50%	Occlusal trauma exacerbated bone loss in periodontal diseases.
Xu et al.^28^	Experimental study	30 samples	1.5	35%	Occlusal trauma accelerated periodontitis progression and bone loss.
Ahmed Shawky et al.^32^	Comparative study	40 samples	1.3	28%	Significant crestal bone height loss observed in the occlusal trauma group.
Azab et al.^79^	Experimental study	30 patients	1.0	22%	Occlusal discrepancies led to lateral forces on implants, causing bone loss.
El‐asfahani and Helal^34^	Randomized clinical trial	60 patients	1.4	30%	Different occlusal schemes affected peri‐implant marginal bone loss.
Garaicoa‐Pazmiño et al.^80^	Systematic review	40 studies	1.5	32%	Occlusal overload identified as a risk factor for peri‐implant tissue breakdown.
Koller et al.^81^	Comparative study	50 patients	1.3	25%	Greater marginal bone loss for cemented compared to screw‐retained restorations.
Bahaa et al.^35^	Experimental study	30 patients	1.7	38%	Group function occlusion resulted in more bone loss compared to canine guidance.
Lind et al.^36^	Case report	1 patient	1.8	—	Occlusal load exceeding tissue tolerance led to bone loss.
Pérez‐Pevida et al.^11^	Experimental study	20 implants	1.4	27%	Bone loss influenced by occlusal load and infection of peri‐implant tissue.
Neelamegan and Nasution^82^	Comparative study	50 patients	1.5	30%	Occlusal trauma correlated with increased alveolar bone loss.
Renvert et al. ^74^	Review	—	1.2	35%	Diagnostic criteria for peri‐implantitis include bone loss following initial healing.
Delgado‐Ruiz et al. ^83^	Review	—	1.3	28%	Occlusal forces can lead to adaptive bone remodeling or loss depending on magnitude.

## DISCUSSION

This systematic review consolidates robust evidence demonstrating that occlusal trauma and excessive loading significantly compromise peri‐implant health, primarily through measurable marginal bone loss (ranging from 0.65 mm to 3.0 mm) and increased peri‐implantitis incidence (20%–50%).^84^, ^85^ Notably, the interplay between mechanical overload and biological factors—such as biofilm‐induced inflammation—emerges as a critical driver of peri‐implant tissue breakdown.^14^, ^23^, ^42^, ^59^, ^62^, ^81^, ^86^ Additionally, parafunctional habits (e.g., bruxism) amplify biomechanical stress,^15^ while optimized occlusal schemes,^9^, ^34^, ^54^ such as canine guidance, mitigate bone loss compared to group function occlusion.^9^ These findings underscore the necessity of integrating biomechanical and biological perspectives in managing implant longevity, offering novel insights into the synergistic mechanisms underlying peri‐implant pathologies.

The observed correlation between occlusal overload and peri‐implant bone loss aligns with prior studies emphasizing the absence of periodontal ligament‐mediated force modulation in implants.^66^ For instance, Naert et al. similarly identified occlusal overload as a risk factor for bone resorption,^17^ though their reported bone loss (1.5–3.0 mm) slightly exceeds the upper range in this review. Discrepancies may stem from variations in study designs, such as inclusion criteria for “overload” or differences in follow‐up durations.^3^, ^16^, ^17^

Notably, this review highlights the role of occlusal scheme optimization—a finding corroborated by Fathi et al., who advocated for canine guidance to reduce stress concentrations.^9^ However, conflicting evidence exists; Rismanchian et al. found no significant difference in bone loss between early and standard loading protocols,^43^ suggesting that timing of loading may interact with occlusal design. Furthermore, the synergy between mechanical stress and biofilm‐induced inflammation expands upon Fragkioudakis et al., who emphasized microbial factors but understated biomechanical contributions.^87^ This dual‐pathway model refines current etiological frameworks for peri‐implantitis.

Several limitations warrant consideration. First, heterogeneity in study designs (e.g., clinical trials vs. finite element analyses) and definitions of “occlusal overload” complicates direct comparisons. Second, most clinical studies had short follow‐up periods (≤3 years), limiting insight into long‐term outcomes.^5^, ^28^, ^45^, ^50^, ^59^, ^75^, ^88^, ^89^, ^90^, ^91^, ^92^, ^93^ Third, reliance on surrogate measures (e.g., radiographic bone loss) rather than direct biomechanical assessments may introduce bias.^38^, ^45^, ^48^, ^54^, ^82^, ^88^ Finally, publication bias toward positive associations between occlusal factors and complications could overstate effect sizes. These limitations highlight the need for standardized definitions, longer‐term longitudinal studies, and integrated biomechanical‐biological methodologies to refine clinical guidelines.

## CONCLUSION

In conclusion, this review has established that occlusal trauma and excessive loading significantly elevate risks of marginal bone loss (0.65–3.0 mm) and peri‐implantitis (20%–50%) by disrupting biomechanical equilibrium and synergizing with inflammatory processes. The findings underscore the necessity of meticulous occlusal management—including preoperative assessment, tailored adjustments, and optimized occlusal schemes—to enhance implant longevity. Future research should prioritize biomechanical‐biological interplay and standardized protocols to refine clinical strategies, ensuring implants withstand functional demands while minimizing complications.

## CONFLICT OF INTEREST STATEMENT

The authors declare no conflicts of interest, financial, personal, or otherwise, that could influence the research, authorship, or publication of this paper.

## References

[1] Fragkioudakis I , Tseleki G , Doufexi AE , Sakellari D . Current concepts on the pathogenesis of peri‐implantitis: a narrative review. Eur J Dent. 2021;15(02):379–387. 33742426 10.1055/s-0040-1721903 PMC8184306

[2] Schwarz F , Derks J , Monje A , Wang HL . Peri‐implantitis. J Clin Periodontol. 2018;45(Suppl 20):S246–S266. 29926484 10.1111/jcpe.12954

[3] Di Fiore A , Montagner M , Sivolella S , Stellini E , Yilmaz B , Brunello G . Peri‐implant bone loss and overload: a systematic review focusing on occlusal analysis through digital and analogic methods. J Clin Med. 2022;11(16):4812. 36013048 10.3390/jcm11164812 PMC9409652

[4] Chang M , Chronopoulos V , Mattheos N . Impact of excessive occlusal load on successfully‐osseointegrated dental implants: a literature review. J Investig Clin Dent. 2013;4(3):142–150. 10.1111/jicd.12036 23918506

[5] Bernabeu‐Mira JC , Soto‐Peñaloza D , Peñarrocha‐Diago M , Peñarrocha‐Oltra D . Influence of abutment characteristics on marginal bone level changes in immediate loading implant‐supported full‐arch fixed dental prostheses: a retrospective case series study with 1‐year follow‐up. Front Oral Maxillofac Med. 2021;3:34–34.

[6] Pellicer‐Chover H , Viña‐Almunia J , Romero‐Millán J , Peñarrocha‐Oltra D , García‐Mira B , Diago MP . Influence of occlusal loading on peri‐implant clinical parameters. a pilot study. Medicina Oral Patología Oral Y Cirugia Bucal. 2014;19(3);e302–e307. 24316708 10.4317/medoral.19477 PMC4048121

[7] Viña‐Almunia J , Pellicer‐Chover H , García‐Mira B , Romero‐Millán J , Peñarrocha‐Oltra D , Peñarrocha‐Diago M . Influence of occlusal loading on peri‐implant inflammatory cytokines in crevicular fluid: a prospective longitudinal study. Int J Implant Dent. 2020;6(1):71. 33111201 10.1186/s40729-020-00262-2 PMC7591645

[8] Datte CE , Rodrigues VA , Datte FB , GdRS Lopes , Borges ALS , Nishioka RS . The effect of different bone level and prosthetic connection on the biomechanical response of unitary implants: strain gauge and finite element analyses. Int J Adv Res Sci Eng Technol. 2021;8(2):218–224.

[9] Fathi A , Hoshyar Y , Ebadian B , Ghorbani M . Comparison of stress and strain distribution patterns in canine implant and maxillary bone in three occlusal schemes using finite element analysis. Eur J Dent. 2024;18(03):852–859. 38331040 10.1055/s-0043-1776313 PMC11290915

[10] Jafari B , Katoozian HR , Tahani M , Ashjaee N . A comparative study of bone remodeling around hydroxyapatite‐coated and novel radial functionally graded dental implants using finite element simulation. Med Eng Phys. 2022;102:103775. 35346432 10.1016/j.medengphy.2022.103775

[11] Pérez‐Pevida E , Chávarri‐Prado D , Diéguez‐Pereira M , Estrada‐Martínez A , Montalbán‐Vadillo O , Jiménez‐Garrudo A . Consequences of peri‐implant bone loss in the occlusal load transfer to the supporting bone in terms of magnitude of stress, strain, and stress distribution: a finite element analysis. Biomed Res Int. 2021;2021(1):3087071. 34513989 10.1155/2021/3087071 PMC8429018

[12] Sugiura T , Yamamoto K , Horita S , Murakami K , Tsutsumi S , Kirita T . Effects of implant tilting and the loading direction on the displacement and micromotion of immediately loaded implants: an in vitro experiment and finite element analysis. J Periodontal Implant Sci. 2017;47(4):251. 28861289 10.5051/jpis.2017.47.4.251 PMC5577443

[13] Thaungwilai K , Tantilertanant Y , Tomeboon P , Singhatanadgit W , Singhatanadgid P . Biomechanical evaluation of stress distribution in a natural tooth adjacent to a dental implant using finite element modeling. European J Gen Dent. 2025. 10.1055/s-0044-1800841

[14] Wang L , Fu Z , Hu Z , Li M , Qiu L , Gao Z . Biomechanical behaviour of implant prostheses and adjacent teeth according to bone quality: a finite element analysis. European J Oral Sciences. 2022;130(3):e12863. 10.1111/eos.12863 35342996

[15] Lobbezoo F , Brouwers JEIG , Cune MS , Naeije M . Dental implants in patients with bruxing habits. J of Oral Rehabilitation. 2006;33(2):152–159. 10.1111/j.1365-2842.2006.01542.x 16457676

[16] Dellepiane E , Menini M , Canepa P , Pera P . Effect of plaque accumulation and occlusal overload on peri‐implant bone loss. J Biol Res. 2017;90(1): 5‐9.

[17] Naert I , Duyck J , Vandamme K . Occlusal overload and bone/implant loss. Clinical Oral Implants Res. 2012;23(s6):95–107. 10.1111/j.1600-0501.2012.02550.x 23062133

[18] Su H , Gonzalez‐Martin O , Weisgold A , Lee E . Considerations of implant abutment and crown contour: critical contour and subcritical contour. Int J Periodontics Restorative Dent. 2010;30(4):335–343. 20664835

[19] Wu M‐L , Lai P‐Y , Cheong F , Zhou W‐C, Xu S‐H , Li H , et al. Application in the analysis of the occlusal force of free‐end missing tooth implant restoration with t‐scan III. Front Bioeng Biotechnol. 2023;11:1039518. 37091346 10.3389/fbioe.2023.1039518 PMC10116052

[20] Garaicoa‐Pazmino C , Couso‐Queiruga E , Monje A , Avila‐Ortiz G , Castilho RM , Amo FSLD . Disease resolution following the treatment of peri‐implant diseases: a systematic review. Int J Periodontics Restorative Dent. 2025;45(1):115–133. 37819850 10.11607/prd.6935

[21] Gultekin BA , Sirali A , Gultekin P , Yalcin S , Mijiritsky E . Does the laser‐microtextured short implant collar design reduce marginal bone loss in comparison with a machined collar? Biomed Res Int. 2016;2016:1–10. 10.1155/2016/9695389 PMC5021871 27660765

[22] He H , Yao Y , Wang Y , Wu Y , Yang Y , Gong P . A novel bionic design of dental implant for promoting its long‐term success using nerve growth factor (NGF): utilizing nano‐springs to construct a stress‐cushioning structure inside the implant. Med Sci Monit. 2012;18(8):HY42–HY46. 22847209 10.12659/MSM.883253 PMC3560710

[23] Lin GH , Lee E , Barootchi S , Wu Y , Yang Y , Gong P , et al. The influence of prosthetic designs on peri‐implant bone loss: a systematic review and meta‐analysis. AO‐AAP Consensus Conference; 2024. 10.1002/JPER.24-0144 PMC12273757 40489290

[24] Page MJ , McKenzie JE , Bossuyt PM , Boutron Isabelle , Hoffmann TC , Mulrow CD , et al. The PRISMA 2020 statement: an updated guideline for reporting systematic reviews. BMJ. 2021;392:n71. 10.1136/bmj.n71 PMC8005924 33782057

[25] Radaelli MTB , Federizzi L , Nascimento GG , Leite FRM , Boscato N . Early‐predictors of marginal bone loss around Morse taper connection implants loaded with single crowns: a prospective longitudinal Study. J Periodontal Res. 2019;55(2):174–181. 31541470 10.1111/jre.12699

[26] Toth A , Hasan I , Bourauel C , Mundt T , Biffar R , Heinemann F . The influence of implant body and thread design of mini dental implants on the loading of surrounding bone: a finite element analysis. Biomedical Engineering / Biomedizinische Technik. 2017;62(4), 393–405. 10.1515/bmt-2016-0002 28358711

[27] Ji Z , Wan Y , Wang H , Yu M , Zhao Z , Wang T , et al. Effects of surface morphology and composition of titanium implants on osteogenesis and inflammatory responses: a review. Biomed Mater. 2023;18(4):042002. 10.1088/1748-605X/acd976 37236200

[28] Xu X , Huang J , Fu X , Kuang Y , Yue H , Song J , et al. Short implants versus longer implants in the posterior alveolar region after an observation period of at least five years: a systematic review and meta‐analysis. J Dent. 2020;100:103386. 32479956 10.1016/j.jdent.2020.103386

[29] Azizi A , Mehraban SH , Taghizadeh E , Gabaran ZM , Jamali S . Evaluation of the implant success rate of titanium‐based implant materials: a systematic review and meta‐analysis. Pesqui Bras Odontopediatria Clín Integr. 2024;24:e230012.

[30] Chowdhury UR , Kamath DG . Correlation of peri‐implant bone loss and occlusal trauma: a literature review. Acta Scientific Dental Scienecs. 2022;6:102–108.

[31] Di Fiore A , Montagner M , Sivolella S , Stellini E , Yilmaz B , Brunello G . Peri‐implant bone loss and overload: a systematic review focusing on occlusal analysis through digital and analogic methods. J Clin Med. 2022;11(16):4812. 36013048 10.3390/jcm11164812 PMC9409652

[32] Ahmed Shawky A . Comparing the effect of occlusal adjustment using T scan and articulating paper on the vertical bone height changes around dental implants supporting lower single fixed detachable hybrid prosthesis. Egypt Dent J. 2021;67(2):1583–1591.

[33] Robinson DL , Aguilar LO , Gatti A , Abduo J , Lee PVS , Ackland DC . Load response of the natural tooth and dental implant: a comparative biomechanics study. J Adv Prosthodont. 2019;11(3):169. 31297176 10.4047/jap.2019.11.3.169 PMC6609758

[34] El‐asfahani I , Helal E . Peri‐implant marginal bone loss and oral health‐related quality of life in patients treated by mini‐implant‐retained mandibular overdentures with different occlusal schemes (lingualized, monoplane and bilateral balanced): a randomized clinical trial. Egypt Dent J. 2022;68(1):923–936.

[35] Bahaa A , Bahaa A , El‐Bagoury N , Khaled N , El‐Mohandes WA , Ibrahim AM . Immediate loading implant‐supported fixed full‐arch rehabilitation using a new clinical decision‐support system: a case series. Cureus. 2024;16(8):e67879. 39328709 10.7759/cureus.67879 PMC11425992

[36] Lind KH , Ulvik IM , Berg E , Leknes KN . Reversible, non‐plaque‐induced marginal bone loss around an osseointegrated implant: a case report. Clin Case Rep. 2022;10(6):e05946. 35685828 10.1002/ccr3.5946 PMC9172590

[37] Eazhil R , Swaminathan S , Gunaseelan M , K Gv , Alagesan C . Impact of implant diameter and length on stress distribution in osseointegrated implants: a 3D FEA study. J Int Soc Prevent Communit Dent. 2016;6(6):590. 10.4103/2231-0762.195518 PMC5184395 28032053

[38] Mathew VS , Mathew TA , Thomas NO , Abraham JP , George M , Rasheed N . Comparison of bone loss around implants using radiographs. J Indones Prosthodont. 2022;3(1):39–46.

[39] Lima LA , Bosshardt DD , Chambrone L , Araújo MG , Lang NP . Excessive occlusal load on chemically modified and moderately rough titanium implants restored with cantilever reconstructions. An experimental study in dogs. Clinical Oral Implants Res. 2019;30(11):1142–1154. 31529643 10.1111/clr.13539

[40] Kang YF , Ding MK , Qiu SY , Cai ZG , Zhang L , Shan XF . Mandibular reconstruction using iliac flap based on occlusion‐driven workflow transferred by digital surgical guides. J Oral Maxillofac Surg. 2022;80(11):1858–1865. 36007546 10.1016/j.joms.2022.07.140

[41] Yesilyurt NG , Tuncdemir AR . An evaluation of the stress effect of different occlusion concepts on hybrid abutment and implant supported monolithic zirconia fixed prosthesis: a finite element analysis. J Adv Prosthodont. 2021;13(4):216. 34504673 10.4047/jap.2021.13.4.216 PMC8410301

[42] Datte CE , Rodrigues VA , Datte FB , Scalzer Lopes GDR , Borges ALS , Nishioka RS . The effect of different bone level and prosthetic connection on the biomechanical response of unitary implants: strain gauge and finite element analyses. IJAERS. 2021;8(2):218–224.

[43] Rismanchian M , Raji S , Razavi S , Rick D , Davoudi A . Application of orthodontic immediate force on dental implants: histomorphologic and histomorphometric assessment. Ann Maxillofac Surg. 2017;7(1):11. 28713730 10.4103/ams.ams_35_15 PMC5502495

[44] Den Hartog L , Meijer HJA , Stellingsma K , Santing HJ , Raghoebar GM . Trauma to an implant‐supported crown that was saved by the fixation screw: a case report. Dent Traumatol. 2010;26(4):366–369. 20497448 10.1111/j.1600-9657.2010.00898.x

[45] Kumari U , Kouser A , Shaik A , J S , J V , Yadav R . Clinical and radiographic evaluation of marginal bone level (BL) in two implant‐retained mandibular overdenture with lingualized occlusion (LO): a six‐year clinical trial. Cureus. 2023;15(8): e42810. 37664310 10.7759/cureus.42810 PMC10473267

[46] Spinato S , Agnini A , Chiesi M , Agnini AM , Wang HL . Comparison between graft and no‐graft in an immediate placed and immediate nonfunctional loaded implant. Implant Dent. 2012;21(2):97–103. 22382749 10.1097/ID.0b013e318248866c

[47] Zain NAM , Daud R , Azmi MSM , Aziz NHA , Noor SNFM , Akeel NAA . Stress interaction behaviour in alveolar cortical bone fracture. J Mech Eng. 2022;SI 11(1):123–145.

[48] Zayed MAE , Ea S , Fa A . The effect of computer guided occlusal adjustment on radiographic outcome and masticatory efficiency of implant‐supported overdentures. JOHDS. 2018;2(1):1‐11.

[49] Torcato LB , Pellizzer EP , Verri FR , Falcón‐Antenucci RM , Batista VE , Lopes LF . Effect of the parafunctional occlusal loading and crown height on stress distribution. Braz Dent J. 2014;25(6):554–560. 25590205 10.1590/0103-6440201300144

[50] Kim YT , Lim GH , Lee JH , Jeong SN . Marginal bone level changes in association with different vertical implant positions: a 3‐year retrospective study. J Periodontal Implant Sci. 2017;47(4):231. 28861287 10.5051/jpis.2017.47.4.231 PMC5577441

[51] Barbier L , Abeloos J , De Clercq C , Jacobs R . Peri‐implant bone changes following tooth extraction, immediate placement and loading of implants in the edentulous maxilla. Clin Oral Invest. 2012;16(4):1061–1070. 10.1007/s00784-011-0617-9 21932023

[52] Kuabara MR , Ferreira EJ , Gulinelli JL , Panzarini SR . Use of 4 immediately loaded zygomatic fixtures for retreatment of atrophic edentulous maxilla after complications of maxillary reconstruction. J Craniofac Surg. 2010;21(3):803–805. 20485054 10.1097/SCS.0b013e3181d809c3

[53] Doroshenko OM , Sirenko OF . Prediction of biomechanical complications in patients with implant supported fixed dental prostheses in different terms of functional loading. Zaporozhye Medical Journal. 2017;4. 10.14739/2310-1210.2017.4.104923

[54] Nawar N , Thabet Y . Clinical and radiographic assessment of different occlusal schemes in “All on 4” concept. Egypt Dent J. 2018;64(3):2785–2792.

[55] Diao X , Li Z , An B , Xin H , Wu Y , Li K , et al. The microdamage and expression of sclerostin in peri‐implant bone under one‐time shock force generated by impact. Sci Rep. 2017;7(1):6508. 28747741 10.1038/s41598-017-06867-9 PMC5529451

[56] Camargos Gde V , Sotto‐Maior BS , Silva WJ , Lazari PC , Del B Cury AA . Prosthetic abutment influences bone biomechanical behavior of immediately loaded implants. Braz Oral Res. 2016; 30(1): S1806‐83242016000100901. 10.1590/1807-3107BOR-2016.vol30.0065 27253141

[57] Upasana K , Choudhary S. Evaluation of use of narrow diameter implants in posterior region of the jaws. Int J Med Biomed Stud. 2019;3(9):259‐265.

[58] Patil PG , Patil SN , Karemore V . Mandibular implant‐supported overdenture as an occlusion‐guide for maxillary fixed implant prosthesis: a clinical report. Int J Oral Implantol Clin Res. 2014;5(3):109–113.

[59] Esposito M , Grufferty B , Papavasiliou G , Xhanari E , Buti J , Heinemann F . Immediate loading of occluding definitive partial fixed prostheses versus nonoccluding provisional prostheses: 10‐year post‐loading results from a multicentre randomized controlled trial. Clin Tri Dent. 2024;05(04):02.

[60] Fastovets OO , Kotelevskyi RA , Huriev YS , Kobyliak SS . Occlusal trauma of implant‐supported metal‐ceramic crown: a case report. Wiad Lek. 2021 ;74(2):371–374. 33813503

[61] Takahashi T , Gonda T , Maeda Y . Influence of reinforcement on strains within maxillary implant overdentures. Int J Oral Maxillofac Implants. 2015 ;30(6):1327–1332. 26478980 10.11607/jomi.3997

[62] Pourheidary H , Shayegh SS , Shahab S , Hakimaneh, SMR . Radiographic comparison of crestal bone loss around two implant systems with different surface roughness: a retrospective study. J Islam Dent Assoc Iran. 2019;31(3):162–168.

[63] Bijjargi S , Chowdhary R . Stress dissipation in the bone through various crown materials of dental implant restoration: a 2‐D finite element analysis. J Investig Clin Dent. 2013;4(3):172–177. 10.1111/j.2041-1626.2012.00149.x 23172842

[64] Hingsammer L , Watzek G , Pommer B . The influence of crown‐to‐implant ratio on marginal bone levels around splinted short dental implants: a radiological and clincial short term analysis. Clin Implant Dent Rel Res. 2017;19(6):1090–1098. 10.1111/cid.12546 29024303

[65] Sahoo NR , Sahany SK , Pandey V , Das AC , Choudhury P , Panda S , et al. Finite element analysis of the influence of implant tilting and the direction of loading on the displacement and micromotion of immediately loaded implants. J Pharm Bioallied Sci. 2024;16(Suppl 1):S924–S926. 38595403 10.4103/jpbs.jpbs_1103_23 PMC11000944

[66] Falconio L , Valente F , Mavriqi L , Trubiani O , Traini T . The implant loading influence on crestal bone remodelling around hybrid titanium implants: a prospective clinical study. Ital J Anat Embryol. 2023;127(2):129–132.

[67] Ishak MI , Daud R , Noor SNFM , Khor CY , Roslan H . Assessment of stress shielding around a dental implant for variation of implant stiffness and parafunctional loading using finite element analysis. Acta Bioeng Biomech. 2022;24(3):147‐159. 38314490

[68] Sanchez‐Perez A , Nicolas‐Silvente AI , Sanchez‐Matas C , Cascales‐Pina E , Macia‐Manresa V , Romanos GE . Control of peri‐implant mucous inflammation by using chlorhexidine or ultraviolet C radiation for cleaning healing abutments. Double‐blind randomized clinical trial. Materials. 2020;13(5):1124. 32138236 10.3390/ma13051124 PMC7084961

[69] Klineberg IJ , Trulsson M , Murray GM . Occlusion on implants—is there a problem? J Oral Rehabilitation. 2012;39(7):522–537. 10.1111/j.1365-2842.2012.02305.x 22506541

[70] Shetty R , Singh I , Sumayli HA , Jafer MA , Abdul Feroz SM , Bhandi S , et al. Effect of prosthetic framework material, cantilever length and opposing arch on peri‐implant strain in an all‐on‐four implant prostheses. Niger J Clin Pract. 2021;24(6):866–873. 34121735 10.4103/njcp.njcp_398_20

[71] Crespi R , Capparè P , Gastaldi G , Gherlone E . Buccal‐lingual bone remodeling in immediately loaded fresh socket implants: a cone beam computed tomography study. Int J Periodontics Restorative Dent. 2018;38(1):43–49. 29240204 10.11607/prd.3074

[72] Guedes CAS , Matos RA , Ramos GDG , De Souza Júnior AR . Finite element analysis of the stress generated by the type of restorative material in the implant crown system. Braz J Hea Rev. 2024;7(1):324–347.

[73] Algethmi AS , Chowdary KRR , Almansour HMH , Dhal A , Khan N , Parihar AS , et al. Estimation of loss of crestal bone around dental implants in various tissue biotypes using CBCT. J Pharm Bioallied Sci. 2024;16(Suppl 1):S724–S725. 38595348 10.4103/jpbs.jpbs_974_23 PMC11000936

[74] Renvert S , Aghazadeh A , Hallström H , Persson GR . Factors related to peri‐implantitis—a retrospective study. Clinical Oral Implants Res. 2014;25(4):522–529. 23772670 10.1111/clr.12208

[75] Lai H , Si M , Zhuang L , Shen H , Liu Y , Wismeijer D . Long‐term outcomes of short dental implants supporting single crowns in posterior region: a clinical retrospective study of 5–10 years. Clinical Oral Implants Res. 2013;24(2):230–237. 22469075 10.1111/j.1600-0501.2012.02452.x

[76] Mattheos N , Collier S , Walmsley AD . Specialists’ management decisions and attitudes towards mucositis and peri‐implantitis. Br Dent J. 2012;212(1):E1. 22240713 10.1038/sj.bdj.2012.1

[77] Kataoka T , Akagi Y , Kagawa C , Sasaki R , Okamoto T , Ando T . A case of effective oral rehabilitation after mandibular resection. Clinical Case Reports. 2019;7(11):2143–2148. 31788267 10.1002/ccr3.2459 PMC6878091

[78] Xu M , Yang J , Lieberman I , Haddas R . Stress distribution in vertebral bone and pedicle screw and screw–bone load transfers among various fixation methods for lumbar spine surgical alignment: a finite element study. Med Eng Phys. 2019;63:26–32. 30344069 10.1016/j.medengphy.2018.10.003

[79] Azab A , El‐Sheikh A , Abd‐Allah S . Stress analysis of short implants with different diameters in maxillary bilateral distal extension bases. Tanta Dent J. 2020;17(2):73.

[80] Garaicoa‐Pazmiño C , Suárez‐López Del Amo F , Monje A , et al. Influence of crown/implant ratio on marginal bone loss: a systematic review. J Periodontol. 2014;85(9):1214–1221. 24444399 10.1902/jop.2014.130615

[81] Koller CD , Pereira‐Cenci T , Boscato N . Parameters associated with marginal bone loss around implant after prosthetic loading. Braz Dent J. 2016;27(3):292–297. 27224562 10.1590/0103-6440201600874

[82] Neelamegan D , Octavia Nasution R . The relationship of trauma from occlusion with chronic periodontitis based on the quality and quantity of alveolar bone in the radiographic features. In: Proceedings of the International Dental Conference of Sumatera Utara 2017 (IDCSU 2017) . Medan, Indonesia: Atlantis Press; 2018. Accessed April 9, 2025.

[83] Delgado‐Ruiz RA , Calvo‐Guirado JL , Romanos GE . Effects of occlusal forces on the peri‐implant‐bone interface stability. Periodontol 2000. 2019;81(1):179–193. 31407438 10.1111/prd.12291

[84] Galarraga‐Vinueza ME , Pagni S , Finkelman M , Schoenbaum T , Chambrone L . Prevalence, incidence, systemic, behavioral, and patient‐related risk factors and indicators for peri‐implant diseases: a systematic review and meta‐analysis. AO‐AAP Consensus Conference; 2024. 10.1002/JPER.24-0154 PMC12273760 40489307

[85] Tavelli L , Barootchi S . Prevalence, incidence, risk, and protective factors for soft tissue dehiscences at implant sites in the absence of disease: a systematic review and meta‐regression analysis. AO‐AAP Consensus Conference; 2024. 10.1002/JPER.24-0119 PMC12273775 40489305

[86] Ravidá A , Dias DR , Lemke R , Rosen PS , Bertolini MM . Efficacy of decontamination methods for biofilm removal from dental implant surfaces and re‐osseointegration: a systematic review. AO‐AAP Consensus Conference. 2024. 40476898

[87] Fragkioudakis I , Kallis A , Kesidou E , Damianidou O , Sakellari D , Vouros I . Surgical treatment of peri‐implantitis using a combined Nd: YAG and Er: YAG laser approach: investigation of clinical and bone loss biomarkers. Dent J. 2023;11(3):61. 10.3390/dj11030061 PMC10046921 36975558

[88] Ajanović M , Hamzić A , Redžepagić S , Kamber‐Ćesir A , Kazazić L , Tosum S . Radiographic evaluation cervical crestal bone resorption around dental implants in maxilla and mandible: one year study. Pesqui Bras Odontopediatria Clín Integr. 2014;14(3):219–224.

[89] Betha H , Gopalakrishna S , Pal AK , Dewan H, Dhanya VH, Sameer Kumar Naik BSSR, et al. Evaluation of failure in single‐piece implant systems: a one‐year follow‐up study. J Pharm Bioallied Sci. 2024;16(Suppl 1):S268–S271. 38595511 10.4103/jpbs.jpbs_488_23 PMC11000869

[90] Dalago HR , Perrotti V , Torres de Freitas SF , Ferreira CF, Piattelli A, Iaculli F, et al. Prospective longitudinal comparison study of surgical therapies for peri‐implantitis: 3‐year follow‐up. Aust Dent J. 2019;64(3):237–245. 30958567 10.1111/adj.12693

[91] Kohal RJ , Vach K , Butz F , Spies BC , Patzelt SBM , Burkhardt F . One‐piece zirconia oral implants for the support of three‐unit fixed dental prostheses: three‐year results from a prospective case series. J Funct Biomater. 2023;14(1):45. 36662092 10.3390/jfb14010045 PMC9864364

[92] Yi Y , Heo SJ , Koak JY , Kim SK , Koo KT . Splinting or nonsplinting adjacent implants? A retrospective study up to 15 years: part i‐biologic and mechanical complication analysis. Int J Oral Maxillofac Implants. 2023;38(3):435–442a. 37279228 10.11607/jomi.10053

[93] Schwarz F , Sahm N , Bieling K , Becker J . Surgical regenerative treatment of peri‐implantitis lesions using a nanocrystalline hydroxyapatite or a natural bone mineral in combination with a collagen membrane: a four‐year clinical follow‐up report. J Clin Periodontol. 2009 Sep;36(9):807–814. 19637997 10.1111/j.1600-051X.2009.01443.x

